# Thoracolaparoscopic radical resection of esophagogastric junction cancers with a NOSE-like approach to extract large specimens

**DOI:** 10.1097/MD.0000000000033120

**Published:** 2023-02-22

**Authors:** Tian-Yu Zhu, Xiu-Mei Deng, Guo-Jun Wang, Jing-Tao Wang, Rui-Xin Li, Bu-Lang Gao, Zhi-Hao Hu

**Affiliations:** a Department of Gastrointestinal Surgery, The First Affiliated Hospital of Zhengzhou University, Zhengzhou, China.

**Keywords:** cancer, esophagogastric junction, laparoscopic, NOSES, thoracoscopic

## Abstract

To investigate the efficacy and safety of combined thoracoscopic and laparoscopic radical resection of esophagogastric junction cancers using a natural orifice specimen extraction-like approach for extraction of large surgical specimens. Patients who had esophagogastric junction cancers treated with thoracolaparoscopic resection using the natural orifice specimen extraction-like approach for specimen extraction were retrospectively enrolled. A 5-cm transverse incision on the abdominal wall at the middle of the superior pubic symphysis was made for surgical specimen extraction. The clinical, surgical, complications, and follow-up data were analyzed. A total of 162 patients were enrolled, and the surgery was successful in all patients (100%). The total surgical duration ranged 165 to 270 minutes, with blood loss 20 to 150 mL, hospital stay 8 to 22 days, first flatus time 2 to 7 days, extubation time of drainage tubes 1 to 26 days, first oral feeding time 5 to 10 days, number of lymph nodes resected 15 to 39, postoperative ambulation time 1 to 2 days, and postoperative residual rate of cancerous cells at the surgical margins 0. Postoperative complications occurred in 14 (8.6%), including anastomotic leakage in 4 (2.5%), anastomotic stenosis in 3 (1.9%), hydrothorax in 4 (2.5%), and incision infection in 3 (1.9%). At follow-up (mean 12 months), all patients were alive, and the transverse incision was a linear scar concealed in the suprapubic pubic hair area. The combined laparoscopic and thoracoscopic surgery for radical resection of carcinomas at the esophagogastric junction is safe and effective, and a transverse incision at the suprapubic symphysis for specimen extraction results in improved minimal invasiveness and cosmesis.

## 1. Introduction

Adenocarcinomas of the esophagogastric junction (EGJ) are tumors that have the lesion center within 2 cm distal or proximal to the anatomical gastric cardia, including three distinct entities: adenocarcinomas of the distal esophagus (1–5 cm from the cardia, Siewert type I), true carcinomas originating from the cardiac epithelium or a short segment with intestinal metaplasia at the EGJ (Siewert type II), and subcardial gastric carcinomas infiltrating the EGJ and distal esophagus from below (Siewert type III).^[1–4]^ The incidence of EGJ adenocarcinomas is increasing rapidly worldwide despite a decreased prevalence of gastric cancers in Europe and North America, possibly because of common etiological causes of obesity and gastroesophageal reflux.^[2,3]^ Curative surgical treatment is the primary approach for resectable EGJ adenocarcinomas with no distant metastasis. Total resection of the cancerous lesion and the entire lymph drainage system with negative surgical margins is conducive to improving the long-term prognosis. Surgical resection approaches of these tumors comprise conventional open surgery and laparoscopic surgery with minimal invasiveness. Laparoscopic gastrointestinal resection is superior to open surgery in clearer visualization, less trauma, less pain, les stress, short hospital stays, excellent cosmetic results, and rapid recovery.^[5–7]^ Recent development in surgical equipment and skills has led to wide acceptance of laparoscopic surgery for gastric submucosal tumors; however, laparoscopic surgery may not be easily applied for EGJ tumors because of technical difficulties and possible postoperative leakage and strictures which may necessitate a further gastrectomy.^[8]^ Nonetheless, laparoscopic surgery has been increasingly performed in patients with EGJ cancers thanks to technical improvement, especially for advanced Siewert type II and type III EGJ carcinomas.^[1,4,5,7,9]^

With the emergence and practice of natural orifice specimen extraction surgery (NOSES), the efficacy and safety of surgical procedures have been greatly facilitated, with further minimization of surgical trauma and postoperative pain but improved cosmetic results.^[10–15]^ The NOSES technique was developed from the totally laparoscopic technique and may represent the next revolution in surgery.^[14,16]^ Natural orifice specimen extraction (NOSE), which avoids extraction-site injury, is defined as the removal of surgical specimens through opening a hollow viscus which communicates with the outside world, including the gastrointestinal tract and vagina. This approach obtains the surgical specimen through a viscerotomy rather than an abdominal incision and avoids the complications related to larger abdominal incisions.^[13]^ Currently, NOSES can be performed in the gastrointestinal, hepatobiliary, urinary, and gynecologic surgery, with four potential natural orifices for specimen extraction, including the anus (rectum), vagina, urethra, and mouth.^[12,14]^ The benefits of NOSES over conventional laparoscopy with abdominal wall specimen extraction include postoperative pain control, shorter time to first bowel function, decreased incisional complications, shorter hospital stay, and improved cosmesis in strictly selected patients.^[13,17–19]^ However, some concerns regarding the NOSES technique have also been raised in the process of specimen extraction, including breakdown of the integrity of the organ used for specimen extraction, infection caused by viscerotomy, pain or functional disturbance of an otherwise healthy organ for specimen extraction, and risk of cancer cell seeding in the organ besides the technical difficulty of NOSES,^[13,20,21]^ which may be amplified by the non-standardized practice of the NOSES technique. Moreover, there is still a steeper learning curve for surgeons to perform viscerotomy for specimen extraction.^[13]^ These issues have prevented wide application of the NOSES technique in abdominal surgery. Therefore, other approaches have to be investigated to improve the minimal invasiveness of these surgeries. In this study, we reported our experience in applying combined thoracoscopic and laparoscopic surgery for radical resection of carcinomas at the EGJ using a NOSE-like approach for extraction of large surgical specimens. In this technique, a small incision was made on the abdominal wall above the symphysis pubis for extraction of large surgical specimens after laparoscopic resection of the tumors, resulting in improved minimal invasiveness and cosmesis but decreased concerns over the NOSES approach through natural orifices for specimen extraction.

## 2. Materials and Methods

### 2.1. Subjects

This retrospective study was approved by the ethics committee of the First Affiliated Hospital of Zhengzhou University, and all patients or their family members had signed the informed consent to participate. Patients with pathologically-confirmed EGJ carcinomas who had undergone endoscopic resection between June 2018 and December 2020 were enrolled. The inclusion criteria were EGJ carcinomas confirmed with endoscopic ultrasound examination and biopsy, at clinically early or progressive stages without distant metastases (T3N2M0), without invading the serosal layer, and without history of esophageal or gastric surgery. The exclusion criteria were patients with tumors with difficulty to be resected, lymph nodes fused to surround important vessels, extensive infiltration of tumor in surrounding tissue unsuitable for complete resection, distant metastases, severe cardiac, pulmonary, hepatic and renal conditions intolerable to surgery, and severe adhesion in chest and abdomen unsuitable for endoscopic surgery.

### 2.2. Surgical procedures

The total procedure was divided into three stages: laparoscopic, NOSES-like, and thoracoscopic surgery. In the laparoscopic surgery, the stomach was dissociated, a gastric tube was constructed, and the thoracic esophagus was mobilized along the fusion fascia plane up to 4 cm above the upper margin of the EGJ cancer for anastomosis of the esophagus with the gastric tube. In the NOSES-like procedure, a transverse incision was made in the middle of the superior pubic symphysis for extraction of a large surgical specimen. In the thoracoscopic surgery, the thoracic esophagus was mobilized and anastomosed with the gastric tube.

### 2.3. Laparoscopic procedure

**Figure 1.**
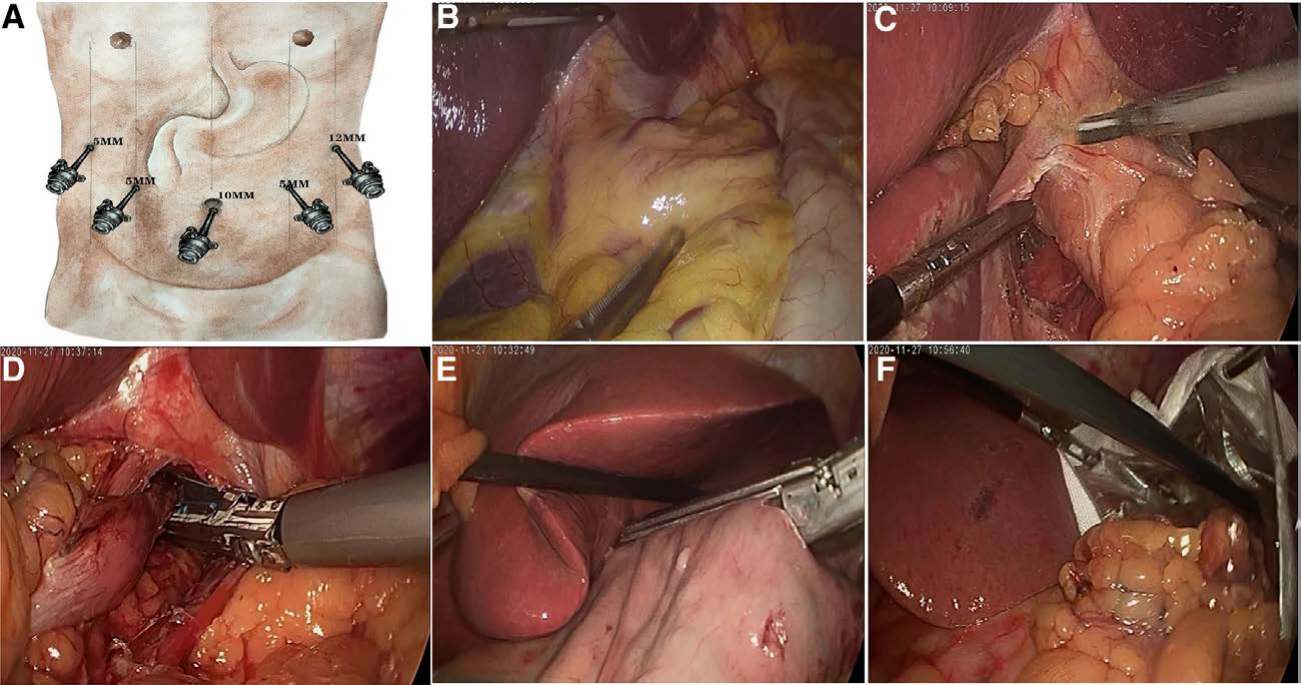
Laparoscopic procedure. A. The location of the five trocars. B. The cancerous lesion was palpated with an instrument. C. The esophagus was mobilized along the fusion fascia plane up to 4 cm above the upper margin of the EGJ cancer. D. The esophagus was cut off for anastomosis of the esophagus with the gastric tube. E. Construction of the gastric tube was begun. F. A specimen bag was sent into the abdominal cavity through the 12 mm main operating hole, and the specimen was put into the bag. EGJ = esophagogastric junction.

The laparoscopic procedures were performed under general anesthesia with the patient in the supine, dorsal elevated position (about 10°) (Fig. 1). Five trocars were placed in the upper abdomen, with a 10 mm trocar 1 cm below the umbilicus as an observation port, a 12 mm trocar for the main operating hole 2 cm below the costal margin of the left anterior axillary line, a 5 mm trocar for auxiliary operation at the intersection of the left middle clavicular line and the horizontal line 2 cm above the umbilicus, a 5 mm trocar 2 cm below the costal margin of the right anterior axillary line, and a 5 mm trocar at the intersection of the right middle clavicular line and the horizontal line 2 cm above the umbilicus. After pneumoperitoneum was established at a pressure of 13 mm Hg, abdominal exploration was performed for possible cancer implantation. Then, the location and size of the cancerous lesion was palpated through an instrument, and the esophagus was mobilized along the fusion fascia plane up to 4 cm above the upper margin of the EGJ cancer and cut off for anastomosis of the esophagus with the gastric tube. After complete resection of the cancerous lesion from 2 cm above the upper margin of the cancerous lesion to 5 cm below the lower margin of the cancer (upper 2/3 of the stomach was resected for Siewert Type I), a gastric tube was constructed from the rest stomach. For patients with Siewert type II or III carcinomas, total gastrectomy was conducted. However, both the thoracoscopic and laparoscopic surgeries were used for all patients in order to ensure sufficient negative margin and sufficient space for operation.

### 2.4. NOSE-like approach for specimen extraction

**Figure 2.**
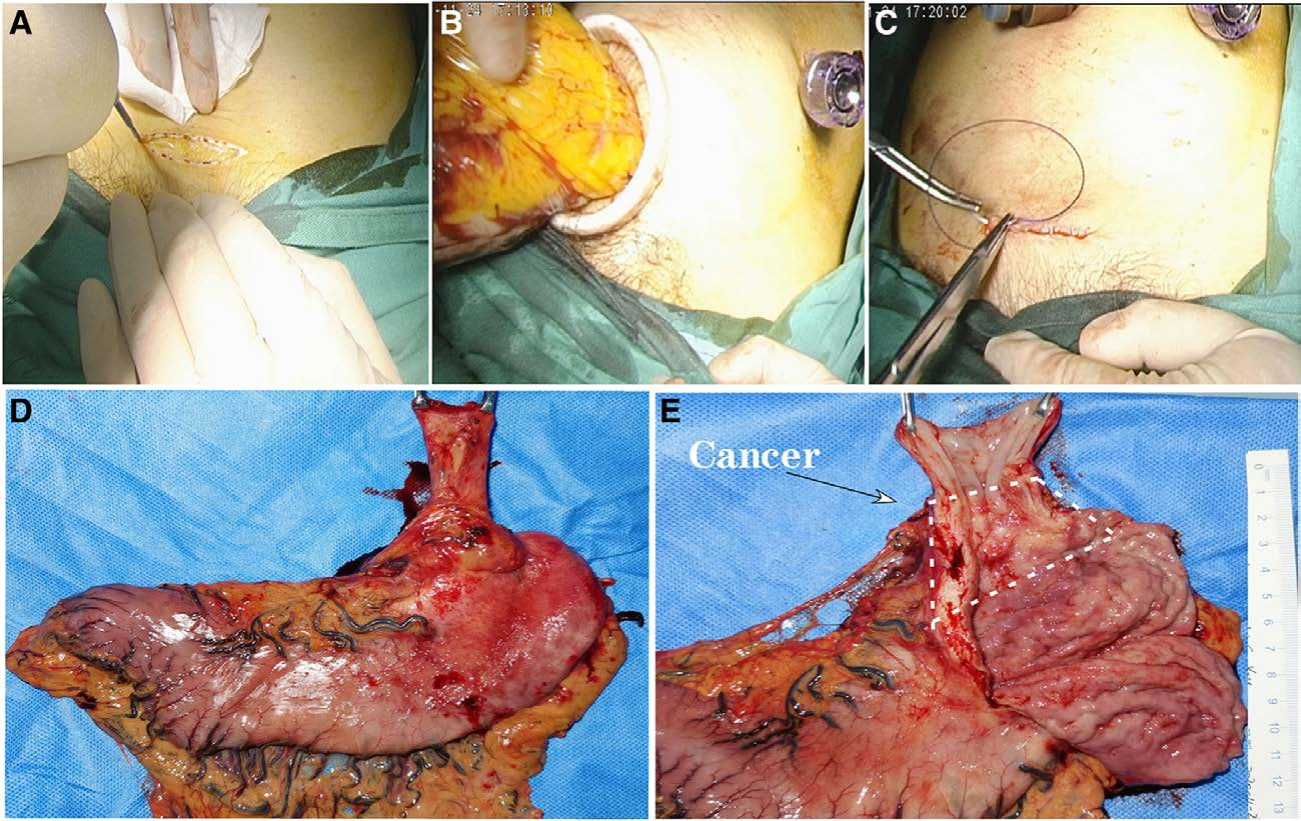
NOSE-like surgery. A. A transverse incision about 5cm long was made in the middle of the superior pubic symphysis. B. The specimen bag was navigated to the lower abdomen and extracted through the transverse incision. C. The incision was closed with absorbable suture to make the scar smaller. D and E. The specimen measured 15 cm × 20 cm including the lower esophagus and the whole stomach. E. The specimen was opened with the cancerous lesion being shown in the white box at the esophagogastric junction. NOSE = natural orifice specimen extraction.

A transverse incision about 5 cm long was made in the middle of the superior pubic symphysis (Fig. 2), and when the anterior sheath of the rectus abdominis was reached, it was incised longitudinally upwards to reach the abdominal cavity. A protective sheath was used to protect the transverse incision. A specimen bag was sent into the abdominal cavity through the 12 mm main operating hole, and the bag mouth was tightened after the whole specimen was put into the bag. Then, the specimen bag was navigated to the lower abdomen and extracted through the transverse incision at the middle of the superior pubic symphysis before subcuticular suturing of the incision with the absorbable suture 4-0. After reestablishment of pneumoperitoneum, the abdominal cavity was washed thoroughly, and the gastric tube was inserted through the esophageal hiatus into the mediastinum, with gastric tube wall fixed to the hiatus muscle to prevent displacement. A drainage tube was placed within the abdominal cavity before closure of the trocar holes.

### 2.5. Thoracoscopic procedure

**Figure 3.**
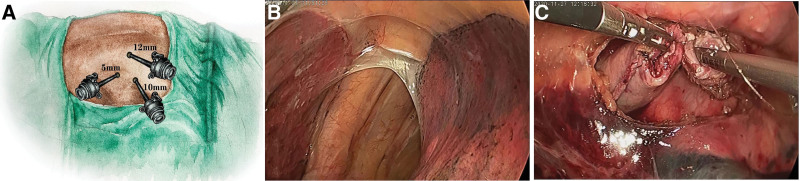
Thoracoscopic procedure. A. The location of three trocars. B. Thoracic exploration was performed for possible adhesion, cancerous implantation, and metastatic lymph nodes. C. The end of the gastric tube was trimmed and anastomosed with the esophagus end to end.

The thoracoscopic procedure was performed with all patients under general anesthesia, and the patient’s position was changed from the supine position in the laparoscopic procedure to the prone position (with the head increased 2° from the operation table and the body increased 2° high on the right side) (Fig. 3). Three trocar holes were made, with a 10 mm trocar for thoracoscopic observation at the intercostal space of the inferior horn of right acromion, a 12 mm trocar for main operation at the fifth intercostal space of the medial border of the right acromion, and a 5 mm trocar hole for observation at the 7th intercostal space 1 cm to the medial side of the 10 mm observation hole. The remaining esophagus after cancer resection was anastomosed to the gastric tube.

### 2.6. Parameters of observation

The duration of surgery, blood loss, postoperative ambulation time, hospital stay, first oral feeding time, number of lymph nodes resected, extubation time of drainage tubes, first flatus time, and incidence of postoperative complications were recorded. The visual analogue scale was used to evaluate the degree of pain within 3 days after operation. Follow-up was performed once every 6 months at the clinics or with telephone, and the survival time was from the time of operation to the last follow-up or death.

### 2.7. Statistical analysis

The statistical analysis was performed using the SPSS software (19.0; IBM, Chicago, IL). Continuous data were presented as mean ± standard deviation, and categorical data as numbers or percentages.

## 3. Results

A total of 162 patients were enrolled, including 105 male and 57 female patients with an age range of 37 to 82 years (median 62) (Table 1). The combined thoracoscopic and laparoscopic surgery was successfully performed in all patients (100%), with no patients being converted to open surgery. Among 162 patients with carcinomas of the EGJ, Siewert type I accounted for 16 cases (9.9%), type II for 85 (52.5%), and type III for 61 (37.7%), including adenocarcinoma in 129 cases (79.6%) and squamous cell carcinoma in 33 (20.4%). The size of tumor ranged 1.5 to 9 cm (mean 5.1) in diameter. The total surgical duration ranged 165 to 270 (mean 210) minutes, with blood loss 20 to 150 mL (mean 82), hospital stay 8 to 22 (mean 12) days, first flatus time 2 to 7 (mean 3.6) days, extubation time of drainage tubes 1 to 26 (mean 7.5) days, first oral feeding time 5 to 10 (mean 7) days, number of lymph nodes resected 15 to 39 (mean 24), postoperative ambulation time 1 to 2 days (mean 1), and postoperative residual rate of cancerous cells at the surgical margins 0. The visual analogue scale scores 24, 48, and 72 hours after the surgery were (2.6 ± 1.0), (2.3 ± 1.1), and (1.6 ± 0.9) at the resting state, respectively, but were (3.1 ± 0.7), (2.8 ± 0.8), and (2.4 ± 1.0) at the active state, respectively. Postoperative complications occurred in 14 (8.6%) patients, including anastomotic leakage in 4 (2.5%), anastomotic stenosis in 3 (1.9%), hydrothorax in 4 (2.5%), and incision infection in three (1.9%). No sequelas were left after proper management.

**Table 1:** Demography and surgical outcomes of patients with gastric cardia carcinoma.

Variables	Data
Patients	
F/M	57/105
Age (yr)	37–82 (median 62)
Cancer	
Siewert type I	16 (9.9%)
Siewert type II	85 (52.5%)
Siewert type III	61 (37.7%)
Size	1.5–9 (mean 5.1)
Histopathology	
Adenocarcinoma	129 (79.6%)
Squamous cell carcinoma	33 (20.4%)
Surgery	
Surgical duration (min)	165–270 (mean 210)
Blood loss (mL)	20–150 (mean 82)
Postoperative data	
Hospital stay (d)	8–22 (mean 12)
Anal exhaust time (d)	2–7 (mean 3.6)
Extubation time of drainage tubes (d)	1–26 (mean 7.5)
First oral feeding time (d)	5–10 (mean 7)
No of lymph nodes resected	15–39 (mean 24)
Postoperative ambulation time (d)	1–2 (mean 1)
Postoperative residual rate of cancerous cells at surgical margins	0
Complications	
Anastomotic leakage	4 (2.5%)
Anastomotic stenosis	3 (1.9%)
Hydrothorax	4 (2.5%)
Incision infection	3 (1.9%)
Follow-up	
Time	2–24 (median 12)
Visible of the transverse scar	Concealed under pubic hair
Death	No

Followed up for 2 months to 2 years (mean 12 months) at clinics or through telephone, all patients were alive, and the transverse incision in the middle of the superior pubic symphysis was a linear scar concealed in the suprapubic pubic hair area.

## 4. Discussion

In this study, radical resection of the EGJ cancers was performed thoracolaparoscopically with a NOSE-like approach to extract large surgical specimens. The small transverse incision in the suprapubic hair area for specimen extraction using the NOSE-like approach will become a linear scar concealed in the suprapubic hair area after healing, with improved cosmesis. This transverse incision can be called an invisible incision without the need of a larger incision on the abdominal wall for extraction of larger specimens. The NOSE-like specimen-extraction approach makes the total thoracolaparoscopic procedure more minimally invasive, and all patients with EGJ carcinomas in this study had been successfully treated this way.

Compared to open transhiatal approach for carcinomas of the EGJ, the laparoscopic approach is associated with significantly shorter postoperative hospital stay (mean difference or MD = −1.70 days), less blood loss (MD, −103 mL), longer operation time (31 minutes, 95% CI 20–41), higher 5-year overall survival rate (risk ratio or RR, 1.43), and significantly improved overall survival, even though the following parameters were not significantly different between the two approaches including the number of harvested lymph nodes (MD, 0.1), time to ambulation (MD, −0.79 days), time to first flatus (MD, −0.82 days), mortality (risk difference or RD, −0.00), total major complication rates (RD, −0.02), and 2-year overall survival rate (RR, 1.17).^[9]^ These outcomes were revealed in a systematic review and meta-analysis on laparoscopic versus open transhiatal surgery for malignancies of EGJ involving nine studies and 2149 patients, indicating the minimal invasiveness and better prognoses of the laparoscopic surgery.^[9]^

In our study, both the thoracoscopic and laparoscopic surgeries were used for radical resection of EGJ cancers, with the esophagus being anastomosed end to end with the reconstructed gastric tube thoracoscopically. The esophagojejunostomy was not used because this approach will probably increase tension of the reconstructed gastrointestinal tract especially for Siewert type I cancers, leading to decreased quality of life. In laparoscopic surgery only, the negative margin of the esophagus may not be ensured, and not sufficient space is available for anastomosis of the esophagus with the gastric tube or jejunum. Moreover, increased tension of the reconstructed gastrointestinal tract may be resulted from esophagojejunostomy. It is why the thoracoscopic surgery was used to ensure sufficient negative margin and sufficient space. The use of gastric tube for anastomosis with the esophagus has ensured free-tension anastomosis but cleared the concern for tensed anastomosis between the esophagus and jejunum.

Traditionally, when laparoscopic gastrectomy was performed, a median incision at the epigastrium was performed, or the trocar incision in the left lower area was extended 4 to 5 cm transversely to retrieve the specimen.^[5]^ In this way, there will be obvious scars or even keloids on the abdominal wall to affect cosmesis. Currently, the NOSES technique has attracted an increasing attention in the world owing to its advantages of minimal post-surgery pain, minimal skin trauma, short hospital stay, positive psychological impact, improved cosmetic effect, and fast recovery.^[13,17–19]^ The NOSES approach has increased the minimal invasiveness of endoscopic surgery, however, for patients with a large specimen especially in the treatment of gastric cancers, the natural orifices like the anus (rectum), vagina, urethra, and mouth are not enough for complete extraction of a larger specimen without functional disturbance of an otherwise healthy organ and risk of cancer cell seeding in the organ besides the technical difficulty of NOSES. In order to retrieve the specimen without an additional incision at epigastrium or without extending a trocar hole, we invented this invisible incision like the NOSE in endoscopic treatment of EGJ carcinomas.

An invisible incision is primarily used in plastic surgery to avoid apparent scars on the skin. In our study, this principle was used for the treatment of EGJ carcinomas using the thoracolaparoscopic surgery. After healing, the transverse incision at the suprapubic symphysis will be a delicate linear scar concealed in the hair area, thus avoiding an apparent scar at the epigastrium. There are some advantages in using this transverse incision at the suprapubic symphysis for specimen extraction. Because the skin at this area is thinner than that in the epigastrium, a scar here is not easy to proliferate, and the incision will be healed like a delicate line which can be concealed by the hair in this area. Moreover, abundant blood supply at this area will speed up the healing of the incision, thus decreasing the risk of infection but shortening the hospital stay. In making this incision at the suprapubic symphysis, stratified incision was performed on the abdominal wall, with the transverse incision in the outer layer but longitudinal incision in the inner layer on the anterior sheath of the rectus abdominis (linea alba) along the skin lines, which will make the scar much smaller after healing. The use of absorbable suture for subcuticular suturing of the incision will leave no apparent scar on the outside abdominal wall.

When resecting the cancerous lesion, a sufficient distance should be ensured for negative margins from the cancerous lesion, and the tumor and lymph nodes within the mesoesophagus and mesogastrium should be resected completely as a whole for radical treatment. All the procedures were performed endoscopically, with only the trocar holes left on the abdominal wall, making the total procedure really minimally invasive. The incision at the suprapubic symphysis for specimen extraction will become a linear scar concealed by the hair. However, this endoscopic surgery is more demanding for endoscopic anastomosis.

In performing a transverse incision at the suprapubic symphysis, the following issues need attention. Because the incision is located suprapubic and close to the bladder, an indwelling catheter should be inserted into the bladder before operation to keep the bladder empty and avoid injury to it. In patients with intestinal adhesion or edema, it will increase the difficulty to extract the specimen and the risk of intestinal injury. Skin preparation is needed for the incision at the suprapubic area. In extraction, the specimen should be avoided to be squeezed to prevent cancerous cell seeding.

Currently, only case reports have been presented in the literature regarding the NOSE approach with laparoscopic radical gastrectomy in malignancies or sleeve gastrectomy in benign lesions, with the specimen being extracted through the mouth, anus or rectum, and vagina.^[14,22–26]^ For extraction of a large specimen, it has to be cut into pieces which had to be stitched to one another before being sutured to a gastric tube and retrieved via the mouth.^[23]^ However, this operation may result in cancerous cell seeding in other locations and metastases. In extracting the specimen through a small orifice or path, the malignant specimen should not be squeezed or cut so as to prevent cancer cell seeding. Natural orifices like the mouth, anus or rectum, and vagina are too small for extracting a large specimen including the lower esophagus and upper stomach. Thus, the invention of an invisible incision at the suprapubic symphysis is necessary to maintain the minimal invasiveness of endoscopic surgery and to extract a large specimen besides improved cosmesis.

Some limitations existed in this study, including the retrospective design, single-center study, Chinese patients enrolled only, no control nor randomization, and short duration of follow-up, which may all affect the generalization of the results. Future prospective, randomized, controlled, multi-center studies involving multiple races will have to solve these issues for better outcomes.

In conclusion, the combined laparoscopic and thoracoscopic surgery for radical resection of carcinomas at the esophagogastric junction is safe and effective, and a transverse incision at the suprapubic symphysis for extraction of large specimens results in improved minimal invasiveness and cosmesis.

## Author contributions

**Conceptualization:** Tian-Yu Zhu, Guo-Jun Wang.

**Data curation:** Tian-Yu Zhu, Xiu-Mei Deng, Jing-Tao Wang, Rui-Xin Li, Zhi-Hao Hu.

**Formal analysis:** Tian-Yu Zhu, Rui-Xin Li, Bu-Lang Gao.

**Funding acquisition:** Guo-Jun Wang.

**Investigation:** Tian-Yu Zhu, Xiu-Mei Deng, Guo-Jun Wang, Jing-Tao Wang, Rui-Xin Li, Zhi-Hao Hu.

**Methodology:** Zhi-Hao Hu.

**Project administration:** Jing-Tao Wang.

**Resources:** Xiu-Mei Deng, Rui-Xin Li, Zhi-Hao Hu.

**Supervision:** Guo-Jun Wang, Rui-Xin Li, Bu-Lang Gao.

**Validation:** Tian-Yu Zhu, Xiu-Mei Deng, Guo-Jun Wang, Jing-Tao Wang, Bu-Lang Gao, Zhi-Hao Hu.

**Visualization:** Xiu-Mei Deng.

**Writing – original draft:** Tian-Yu Zhu.

**Writing – review & editing:** Bu-Lang Gao.
